# A composite biomarker based on early dynamic changes in lactate dehydrogenase and PD-L1 expression enhances outcome prediction for immunochemotherapy in HER2-negative advanced gastric cancer

**DOI:** 10.3389/fimmu.2026.1659007

**Published:** 2026-02-12

**Authors:** Yue Zhang, Yang Tian, Xiaoli Wei, Lu Bai, Meiqi Song, Yuanzhi Guo, Tianlai Liu, Xing Li, Weiqi Shan, Huaxing Wu, Yu Han

**Affiliations:** 1 Department of Gastrointestinal Medical Oncology, Harbin Medical University Cancer Hospital, Harbin, Heilongjiang, China; 2 Department of Endoscopy, Harbin Medical University Cancer Hospital, Harbin, Heilongjiang, China; 3 Affiliated Cancer Hospital of Inner Mongolia Medical University, Hohhot, Inner Mongolia, China

**Keywords:** combined positive score, composite biomarker, dynamic monitor, gastric cancer, immunochemotherapy, lactate dehydrogenase

## Abstract

**Introduction:**

Current biomarkers for immunochemotherapy in HER2-negative advanced gastric cancer (AGC) exhibit inadequate predictive accuracy. Therefore, the development of innovative methodologies is imperative for improving patient selection.

**Methods:**

In this retrospective biomarker study, a cohort of 107 patients with HER2-negative AGC undergoing first-line immunochemotherapy was examined. Serum biomarkers were serially assessed at baseline and after two treatment cycles. Univariate and multivariate Cox regression analyses, as well as ROC curve analysis, were employed to identify prognostic serum dynamics. A composite biomarker integrating dynamic changes in lactate dehydrogenase (LDH) and a combined positive score (CPS) was developed and validated. Additionally, exploratory genomic profiling was performed.

**Results:**

In this study, dynamic changes in LDH were identified as an independent prognostic factor. An increase in LDH of ≥26% after two treatment cycles was associated with significantly poorer progression-free survival (PFS: 5.33 vs. 8.93 months; HR: 2.585, P< 0.001) and overall survival (OS: 13.13 vs. 19.63 months; HR: 2.689, P< 0.001). Consistent results were observed in landmark analyses at the two-cycle timepoint, with significant differences in PFS-landmark (PFS-landmark: 5.17 vs. 8.40 months; HR: 2.450, P< 0.001) and OS-landmark (OS-landmark: 11.76 vs. 19.20 months; HR: 2.538, P< 0.001). Combining LDH dynamics with baseline CPS overcame the predictive limitations observed in patients with CPS<5. The subgroup within the composite biomarker (baseline CPS<5 plus LDH elevation ≥26% after two cycles) demonstrated poor prognosis and high risk of early disease progression in survival analysis (median PFS: 4.43 months; median OS: 12.26 months). The robustness of this biomarker was supported by ROC and calibration curves. Additionally, exploratory genomic analysis suggested potential prognostic implications of specific KRAS mutant subtypes and co-mutation patterns. However, validation of these findings is warranted through prospective, multi-center-controlled cohort studies due to constraints related to sample size.

**Conclusion:**

In HER2-negative advanced gastric cancer, a ≥26% increase in LDH following two treatment cycles serves as an independent prognostic risk factor. Combining baseline CPS with early LDH dynamics improves the identification of patients with aggressive disease and poor initial treatment response, thereby facilitating closer monitoring and prompt therapeutic reassessment for more personalized management.

## 1 Introduction

Gastric cancer poses a significant global health challenge, being the fifth most diagnosed cancer and the fourth leading cause of cancer-related deaths worldwide. Annually, nearly one million new cases are reported, with an incidence rate of 5.6%. The disease accounts for over 650, 000 deaths each year, representing a mortality rate of 7.7% (1). The poor prognosis of advanced gastric cancer (AGC) is primarily attributed to frequent late-stage diagnosis and limited treatment modalities. The integration of immune checkpoint inhibitors (ICIs) with chemotherapy has redefined first-line treatment for human epidermal growth factor receptor 2 (HER2)-negative AGC; however, substantial variability in treatment response among patients persists (2, 3). Currently, patient selection for immunochemotherapy primarily hinges on the evaluation of baseline programmed death ligand 1(PD-L1) expression in tumor biopsy samples. This approach is encumbered by challenges such as spatial and temporal heterogeneity in PD-L1 expression, lack of standardized detection methods, and absence of consensus on the optimal cutoff value for PD-L1 expression (4). These obstacles underscore the critical necessity for novel and practical composite biomarkers that can reliably predict treatment responses.

Theoretically, continuous molecular profiling of tumor tissue could address the evolving microenvironmental changes during therapy, including immune cell functional alterations and metabolic reprogramming (5–7). However, the practical application of sequential biopsies in clinical settings is hindered by procedural invasiveness, financial constraints, and sampling limitations. Novel liquid biopsy methodologies, particularly monitoring circulating tumor DNA (ctDNA), exhibit promising associations with treatment efficacy but encounter technical and economic barriers that impede routine integration (8). In contrast, blood-based biomarker assays present enhanced clinical feasibility and practicality. Increasing evidence indicates that peripheral blood biomarkers reflecting tumor burden or systemic inflammation, such as the neutrophil-to-lymphocyte ratio (NLR), platelet-to-lymphocyte ratio (PLR), prognostic nutritional index (PNI), and platelet-to-neutrophil ratio (PNR), hold potential as predictors of immunotherapy outcomes (9–12). Nevertheless, existing studies have predominantly focused on static baseline levels of these biomarkers, while the relationship between dynamic alterations in peripheral blood parameters during treatment and clinical results remains inadequately characterized.

Consequently, the primary objective of this study is to utilize longitudinal assessment of blood biomarkers to discover predictive molecular profiles that can enhance the predictive value of PD-L1 expression. Through this approach, our aim is to determine the populations that are prone to resistance to immunotherapy and have an advantage, thereby optimizing the diagnosis and treatment strategies for specific patient groups.

## 2 Materials and methods

### 2.1 Study population

This retrospective investigation was conducted at Harbin Medical University Cancer Hospital and involved the recruitment of patients diagnosed with advanced gastric adenocarcinoma through histological examination. The study enrolled patients who commenced first-line ICIs combined with chemotherapy between September 18, 2020, and May 15, 2023. Inclusion criteria comprised: (i) patients with histologically confirmed unresectable advanced gastric adenocarcinoma; (ii) patients receiving immunochemotherapy as their initial treatment; (iii) Eastern Cooperative Oncology Group (ECOG) performance status of 0 or 1; and (iv) patients with confirmed HER2-negative status. Exclusion criteria encompassed: (i) recent administration of anticancer therapy or medications affecting hematological parameters within 4 weeks before enrollment; (ii) presence of other active malignancies or a history of concurrent hematologic disorders; (iii) incomplete baseline clinical and pathological data; (iv) life expectancy of less than 3 months; and (v) patients who were lost to follow-up.

Patients were treated with ICIs combined with chemotherapy. The detailed treatment regimens are outlined in **Supplementary Table 1**. All patients underwent a minimum of two cycles of combined therapy. Evaluation of treatment effectiveness was conducted 6–8 weeks after treatment initiation and was subsequently monitored at regular intervals.

#### 2.1.1 Data collection

Patient data, clinical variables, and laboratory results were extracted from the electronic medical record system. Peripheral blood biomarkers were analyzed at baseline (within 7 days before treatment) and after two treatment cycles (within 6–8 weeks post-treatment). The longitudinal changes in biomarkers were quantified utilizing the following formula:


$$\text{Perentage change}=\frac{\left(\text{Post}-\text{treatment value}-\text{Baseline value}\right)}{\text{Baseline value}}\times 100\%$$

Details of blood parameters are provided in **Supplementary Tables 2** and **3**.

#### 2.1.2 Detection methods of molecular biomarkers

For biomarker detection using untreated tumor tissue, a minimum of three biopsy samples or complete tissue sections from gastrectomy specimens were collected based on tumor characteristics such as size and location. These samples were evaluated independently by two pathologists. In case of disagreement, a third senior pathologist was consulted to arbitrate and establish the final immunohistochemistry (IHC) result. The evaluation of HER2 status involved the utilization of both IHC and fluorescence *in situ* hybridization (FISH). HER2 expression via IHC staining was categorized into a four-tier grading system for gastric adenocarcinoma: IHC 0 (no staining or membrane staining in<10% of tumor cells), IHC 1+ (faint or barely perceptible membrane staining in ≥10% of tumor cells), IHC 2+ (weak to moderate complete or basolateral membrane staining in ≥10% of tumor cells), and IHC 3+ (moderate to strong complete or basolateral membrane staining in ≥10% of tumor cells). Tumors with an IHC score of 2+ underwent FISH analysis to confirm HER2 gene amplification. HER2 positivity was defined as either IHC 3+ or IHC 2+ with FISH-positive gene amplification. Samples exhibiting IHC 0 or 1+, or IHC 2+ without FISH-confirmed gene amplification, were considered HER2-negative (13). This study exclusively enrolled HER2-negative patients, who were further categorized based on IHC staining intensity into HER2-low (IHC 1+, or IHC 2+) and HER2-null (IHC 0) subgroups.

The assessment of mismatch repair (MMR) status and microsatellite instability (MSI) was conducted through IHC analysis of MMR protein expression (MLH1, PMS2, MSH2, MSH6) or via next-generation sequencing (NGS) utilizing formalin-fixation and paraffin-embedding (FFPE) tumor tissue specimens. PD-L1 composite positive score (CPS) was determined using immunohistochemistry with the Dako 22C3 antibody. CPS was defined as the ratio of the number of PD-L1-positive tumor cells (with partial or complete membrane staining), lymphocytes, and macrophages (with membrane staining, cytoplasmic staining, or both) to the total number of viable tumor cells, multiplied by 100. Targeted genomic sequencing was performed on tumor tissues and matched peripheral blood samples using GeneseeqPrime™437 and YuceOne™1012 gene panels, featuring a consensus gene panel of 364 genes for analysis.

### 2.2 Efficacy evaluation and follow-up

Treatment efficacy was evaluated based on RECIST v1.1 criteria and categorized as complete response (CR), partial response (PR), stable disease (SD), or progressive disease (PD). The objective response rate (ORR) was calculated as the proportion of patients achieving CR or PR, while the disease control rate (DCR) included patients with CR, PR, or SD. Follow-up assessments were conducted every 2–3 months through outpatient visits or telephone interviews. Progression-free survival (PFS) was measured from the start of immunochemotherapy to the occurrence of radiographic or clinical progression or death, whichever came first; for patients without progression, data were censored at the time of the last follow-up. Overall survival (OS) was calculated from the initiation of immunochemotherapy to death or the last follow-up.

For landmark analysis, the time point corresponding to lactate dehydrogenase (LDH) level assessment after two treatment cycles was selected as the landmark. PFS and OS measured from this landmark were designated as PFS-landmark and OS-landmark, respectively. To minimize the potential for immortal time bias in the landmark analysis, only patients who were alive and progression-free after the second treatment cycle were included.

### 2.3 Statistical analysis

Statistical analyses were handled utilizing SPSS 26.0 and GraphPad Prism 9.0. Patient demographics and clinical characteristics were presented as absolute frequencies along with their corresponding percentages. Group comparisons for categorical variables were conducted using the chi-square test or Fisher’s exact test, depending on the data distribution. The relationship between the percentage change in LDH levels after treatment and survival rates at different time points was assessed by constructing receiver operating characteristic (ROC) curves. The optimal cutoff value was determined based on the ROC curve for 12-month survival, which was identified as ≥26%. To evaluate the robustness of this cutoff value, additional thresholds (≥21%, ≥24%, ≥28%, and ≥31%) were tested in subsequent survival analyses. PFS and OS were analyzed using the Kaplan-Meier method, with intergroup differences assessed via the log-rank test. Univariate Cox regression analysis was used to calculate the hazard ratio (HR) and its corresponding 95% confidence interval (CI). Multivariate survival analysis was conducted using multivariate Cox regression. Furthermore, the discriminative ability of combined biomarkers was assessed using ROC curves and calibration curves. All statistical tests were two-sided, with statistical significance set at P< 0.05.

## 3 Results

### 3.1 The characteristics and survival of the overall population

**Figure 1:**
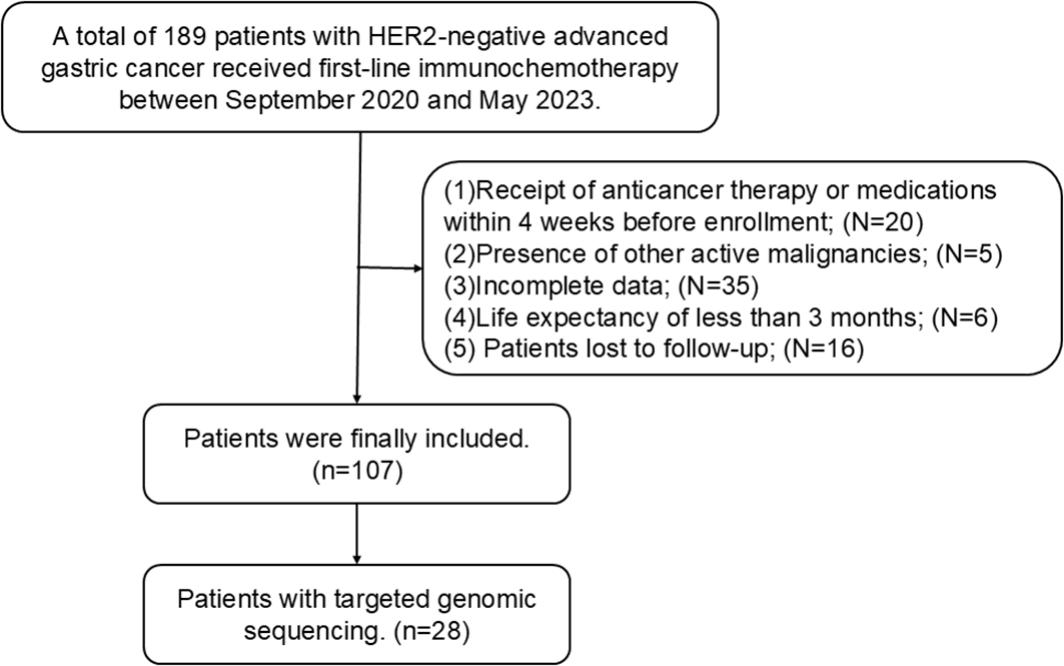
Flowchart of the screening process for the research population.

This study enrolled 107 patients with advanced gastric cancer (**Figure 1**). The majority of patients (69%) were under the age of 65. Among the cohort, 32 patients were female (30%) and 75 were male (70%). The majority of patients had poorly differentiated carcinoma (69%), 42 patients had a history of gastrectomy (39%), 78 patients had lymphatic metastasis (73%), 33 patients had liver metastasis (31%), and 46 patients had peritoneal metastasis (43%). Regarding the choice of combination chemotherapy regimen, 92 patients (86%) received XELOX/SOX (Oxaliplatin +Capecitabine/Oxaliplatin+S-1), while 15 patients (14%) were treated with AS(Nab-paclitaxel+S-1). Detailed baseline characteristics are detailed in **Table 1**.

**Table 1:** Baseline characteristics.

Variables	Total (n = 107)	Variables	Total (n = 107)
	No. (%)		No. (%)
**Age**		**Chemotherapy regimens**	
<65	74 (69)	SOX/XELOX	92 (86)
>=65	33 (31)	AS	15 (14)
**Gender**		**Second-line**	
Female	32 (30)	No	42 (39)
Male	75 (70)	Yes	65 (61)
**ECOG**		**HER2 expression**	
0	28 (26)	HER2-null	67(52)
1	79 (74)	HER2-low	40(31)
**BMI**		**CPS**	
<23	66 (62)	>=5	28 (26)
>=23	41 (38)	<5	43 (40)
**Gastrectomy**		Unknown	36 (34)
No	65 (61)	**MSI status**	
Yes	42 (39)	MSS/pMMR	46 (43)
**Tumor location**		MSI-H/dMMR	5 (5)
GEJ	6 (6)	Unknown	56 (52)
Non-GEJ	101 (94)	**Response**	
**Tumor differentiation**		PR	26
Well-moderate	8 (7)	SD	38
Poor	74 (69)	PD	9
Unknown	25 (23)	**ORR**	35.61%
**Lymph metastasis**	78 (73)	**DCR**	87.67%
**Liver metastases**	33 (31)		
**Peritoneal metastasis**	46 (43)		
**The anti-PD1 drug**			
Sintilimab	52(49)		
Camrelizumab	40(37)		
Toripalimab	7(7)		
Pembrolizumab	5(5)		
Nivolumab	3(2)		

ECOG, Eastern Cooperative Oncology Group; BMI, Body mass index; GEJ, Gastroesophageal junction; XELOX/SOX, Oxaliplatin +Capecitabine/Oxaliplatin+S-1; AS, Nab-paclitaxel+S-1; HER2,Human epidermal growth factor receptor 2; CPS, Composite positive score; MSI, Microsatellite instability; MMR, Mismatch repair; MSS, MSI-low/microsatellite stability; pMMR, Proficient MMR; MSI-H, MSI-high; dMMR, Deficient MMR; LDH, Lactate dehydrogenase; ULN, Upper limit of normal; NLR, Neutrophil to lymphocyte ratio; PR, Partial response; SD, Stable disease; PD, Progressive disease; ORR, Objective response rate; DCR, Disease control rate. The bold values represent the definitions of variables.

The individuals were monitored until 30 July 2024. The median follow-up duration was 31.63 months (95% CI: 28.45-34.82). The median PFS and OS for the overall population were 8.3months (95% CI: 7.04-9.56) and 16.17 months (95% CI: 13.92-18.41), respectively. Initially, 73 patients had at least one target lesion, with 26 showing PR, 38 SD, and 9 PD, resulting in an ORR of 35.61% and a DCR of 87.67% (**Table 1**).

### 3.2 The role of post-treatment changes in peripheral blood parameters to predict treatment outcome

**Figure 2:**
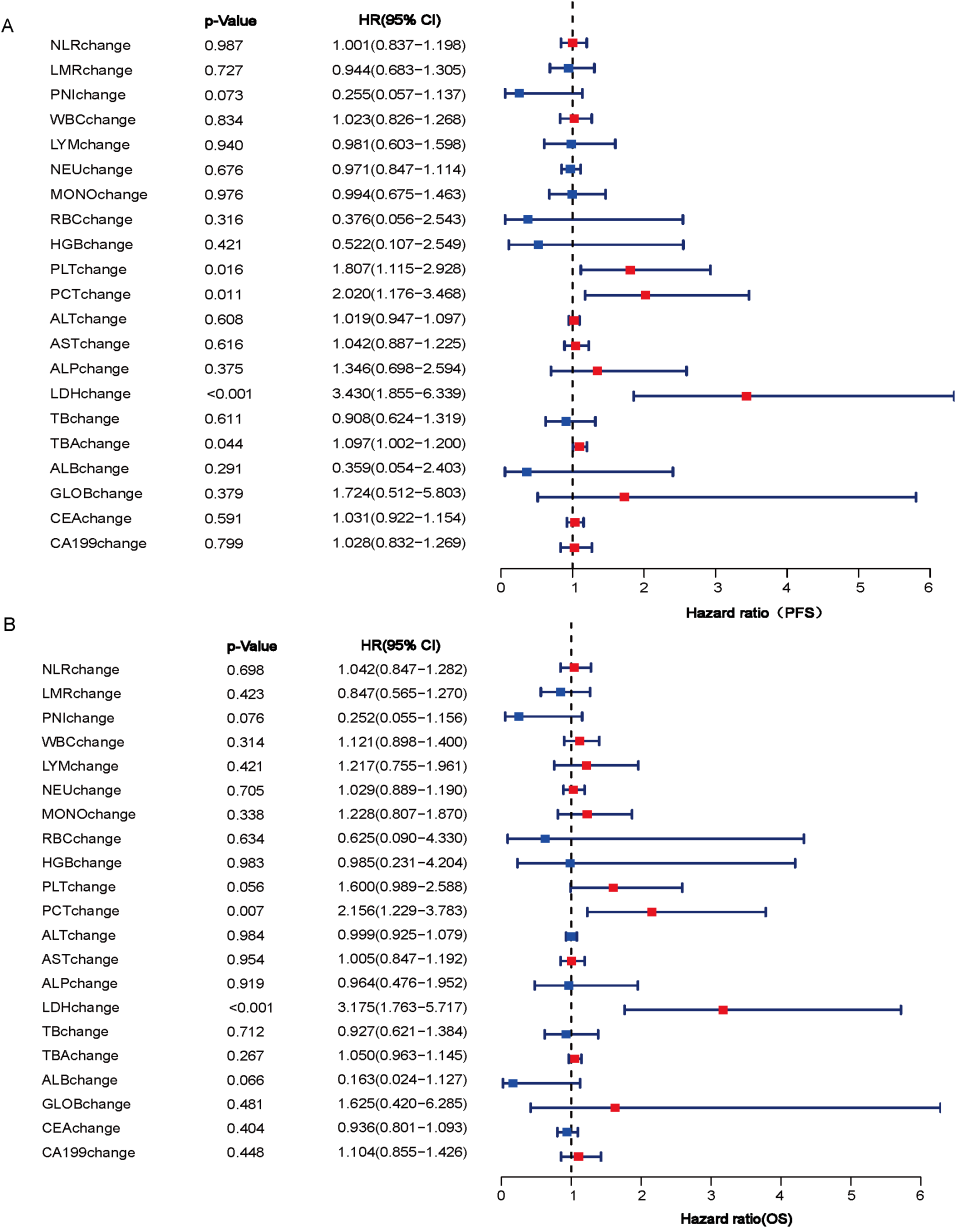
Association of post-treatment changes in peripheral blood parameters with PFS **(A)** and OS **(B)**. NLR, neutrophil-to-lymphocyte ratio; LMR, lymphocyte-to-monocyte Ratio; PNI, prognostic nutritional index; WBC, white blood cell; LYM, lymphocyte; NEU, neutrophil; MONO, monocyte; RBC, red blood cell; HGB, hemoglobin; PLT, platelets; PCT, platelet crit; ALT, alanine transaminase; AST, aspartate transaminase; ALP, alkaline phosphatase LDH, lactate dehydrogenase; TB, total bilirubin; TBA, total bile acid; ALB, albumin; GLOB, globulin; CEA, carcinoembryonic antigen; CA199,carbohydrate antigen 199; HR, hazard ratio; CI, confidence intervals; PFS, progression-free survival; OS, overall survival.

In our investigation, we explored the relationship between post-treatment alterations in peripheral blood parameters and the prognosis of patients undergoing a combination of immunotherapy and chemotherapy. Univariate Cox regression analyses revealed significant associations between changes in platelet count (PLT), platelet crit (PCT), LDH, and total bile acid (TBA) with PFS (P<0.05), while PCT and LDH changes were significantly linked to OS (P<0.05) (**Figure 2**). Subsequent multivariate Cox regression analysis confirmed that post-treatment changes in LDH were independently predictive of both PFS and OS (**Table 2**). These findings highlight the potential of LDH as a valuable biomarker for assessing treatment outcomes in patients with advanced gastric cancer undergoing a combination of immunotherapy and chemotherapy.

**Table 2:** Multivariate analysis of changes in peripheral blood parameters for PFS and OS.

	PFS		OS	
	HR (95%CI)	P value	HR (95%CI)	P value
PLT-change	1.064(0299-3.789)	0.924	0.395(0.113-1.378)	0.145
PCT-change	1.788(0.425-7.528)	0.428	4.978(1.213-20.426)	0.026
LDH-change	3.05(1.612-5.768)	0.001	2.907(1.590-5.313)	0.001
TBA-change	1.08(0.985-1.184)	0.101	1.012(0.928-1.103)	0.788

PLT, platelets; PCT, platelet crit; LDH, Lactate dehydrogenase; TBA, Total bile acid; PFS, progression-free survival; HR, Hazard ratios; CI, confidence intervals; OS, overall survival.

### 3.3 The role of post-treatment changes in LDH levels to predict treatment outcome

**Figure 3:**
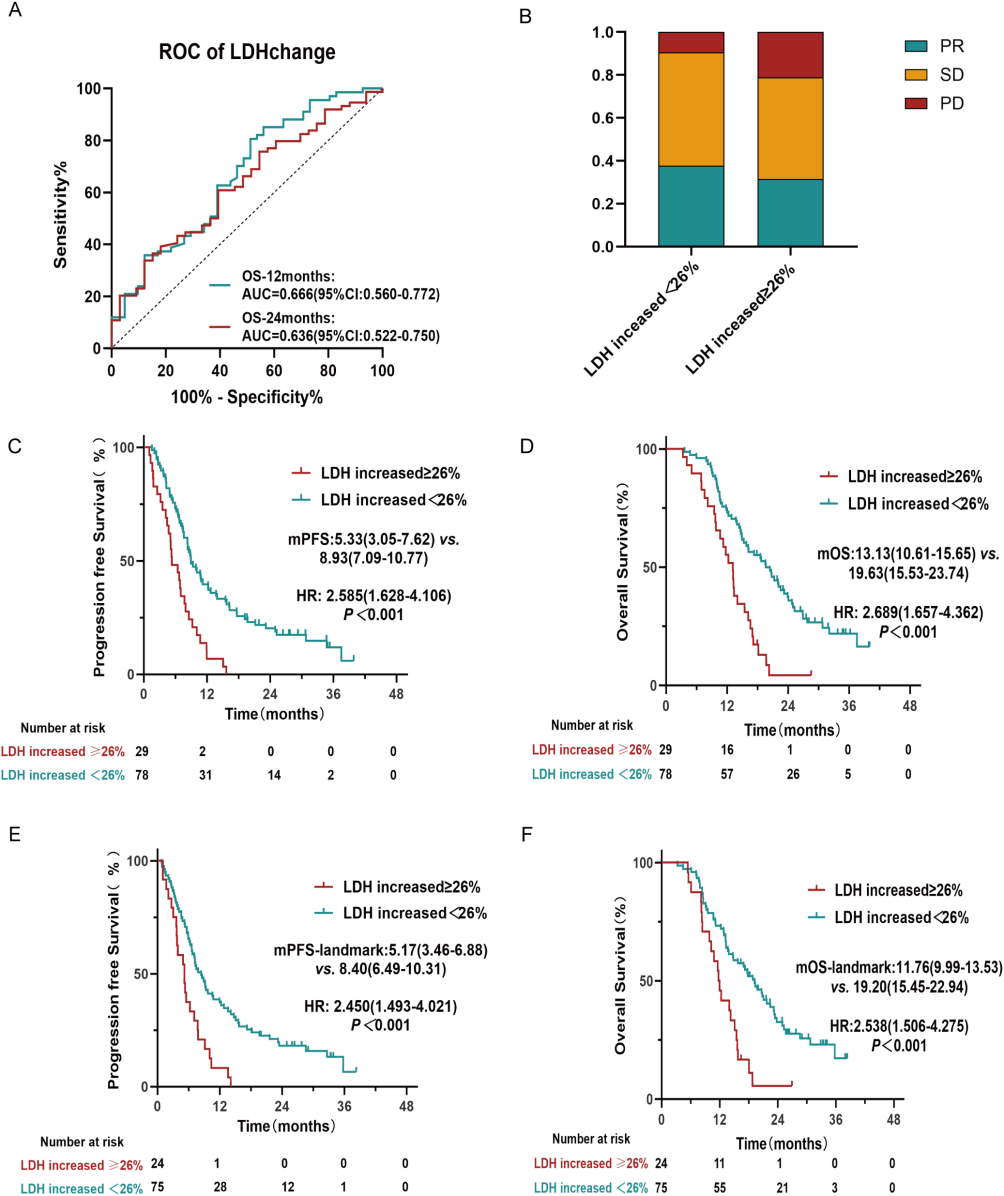
Prognostic significance of post-treatment LDH changes. **(A)** ROC curves of LDH change as a prognostic marker for overall survival (OS) at 12 months and 24 months. **(B)** Distribution of treatment response (PR, SD, PD) stratified by post-treatment LDH increased ≥26% versus<26%. **(C, D)** Progression-free survival (PFS) and Overall survival (OS) in patients with post-treatment LDH increased by≥26% versus<26%. **(E, F)** PFS-landmark and OS-landmark in patients with post-treatment LDH increased by≥26% versus<26%.

Using ROC curve analysis, we determined that a 26% increase in post-treatment LDH levels was the optimal cut-off value (**Figure 3A**). Notably, the AUC for OS at 12 months was 0.666(95%CI:0.560-0.772). To evaluate the robustness of this threshold, we performed a sensitivity analysis across a range of LDH increases (21% to 31%). Log-rank tests consistently yielded p-values below 0.05 within this range, indicating the continued statistical significance of the association with survival and supporting the stability of the 26% cut-off (**Supplementary Table 4**). The clinicopathological characteristics did not display significant differences between these cohorts (**Supplementary Table 5**). There were no significant differences in DCR (90.74% vs. 78.95%, P = 0.179) and ORR (37.03% vs. 31.58%, P = 0.669) between the two cohorts (**Figure 3B**). However, patients with an increase in LDH ≥26% demonstrated a median PFS of 5.33 months, compared with 8.93 months in those with an LDH increase<26% (HR: 2.585, 95% CI: 1.628–4.106, P< 0.001) (**Figure 3C**). Similarly, median OS was 13.13 months versus 19.63 months, respectively (HR: 2.689, 95% CI: 1.657–4.362, P< 0.001) (**Figure 3D**). Furthermore, landmark analysis yielded results consistent with the conventional analyses for both PFS and OS (P< 0.05). In the landmark analysis, only patients who were alive and progression-free after the second treatment cycle were included(n=99). The median PFS-landmark was 5.17 months in patients with an LDH increase ≥26%, compared to 8.40 months in those with an LDH increase<26% (HR: 2.450, 95% CI:1.493-4.021, P< 0.001) (**Figure 3E**). The corresponding median OS-landmark was 11.76 months versus 19.20 months (HR: 2.538, 95% CI: 1.506-4.275, P< 0.001) (**Figure 3F**). These findings support the robustness of our results.

### 3.4 Prognostic factor analysis

In the univariate analysis, female, PD-L1 CPS<5, and a post-treatment increase in LDH levels ≥26% were correlated with unfavorable outcomes in both PFS and OS. Multivariate Cox regression analysis confirmed that these variables were independent predictive factors for both PFS and OS (**Tables 3**, **4**). In Model 2, which included additional variables such as lymph node metastasis, liver metastasis, peritoneal metastasis, chemotherapy regimen, and second-line therapy, sex, PD-L1 CPS, and post-treatment LDH change remained significant predictors of PFS and OS, while other variables showed no significant association.

**Table 3:** Univariate and multivariate analysis of biomarkers for PFS.

Variables	Univariate analysis	*P*	Model 1	*P*	Model 2	*P*
	HR(95%CI)		HR(95%CI)		HR(95%CI)	
Age						
>=65	Ref					
<65	1.090(0.698-1.701)	0.705				
Gender						
Male	Ref					
Female	1.905(1.231-2.948)	0.004	2.842(1.638-4.934)	<0.001	3.305(1.753-6.231)	<0.001
ECOG						
0	Ref					
1	1.060(0.675-1.664)	0.802				
BMI (kg/m2)						
>=23	Ref					
<23	1.124(0.745-1.697)	0.578				
Gastrectomy						
No	Ref					
Yes	1.153(0.763-1.742)	0.5				
Tumor location						
Non-GEJ	Ref					
GEJ	1.716(0.742-3.969)	0.207				
Tumor differentiation						
Poor	Ref					
Well-moderate	1.069(0.512-2.231)	0.86				
Lymph metastasis						
Yes	Ref					
No	1.088(0.692-1.710)	0.714			2.041(1.066-3.908)	0.031
Liver metastasis						
Yes	Ref					
No	1.055(0.684-1.628)	0.809			1.131(0.639-2.001)	0.673
Peritoneal metastasis						
No	Ref					
Yes	1.322(0.881-1.984)	0.177			0.684(0.372-1.259)	0.222
Chemotherapy regimens						
SOX/XELOX	Ref					
AS	1.108(0.627-1.957)	0.724			0.976(0.459-2.078)	0.951
HER2 status						
Null	Ref					
Low	1.239(0.821-1.869)	0.309				
MSI status						
MSI-H/pMMR	Ref					
MSS/dMMR	2.095(0.646-6.796)	0.218				
CPS						
>=5	Ref					
<5	1.993(1.183-3.356)	0.01	1.773(1.048-2.999)	0.033	2.098(1.192-3.692)	0.01
LDH						
<=ULN	Ref					
>ULN	1.413(0.764-2.613)	0.271				
LDH increased						
<26%	Ref					
>=26%	2.585(1.628-4.106)	<0.001	5.073(2.633-9.773)	<0.001	3.305(1.753-6.231)	<0.001

ECOG, Eastern Cooperative Oncology Group; BMI, Body mass index; GEJ, Gastroesophageal junction; XELOX/SOX, Oxaliplatin +Capecitabine/Oxaliplatin+S-1; AS, Nab-paclitaxel+S-1; HER2,Human epidermal growth factor receptor 2; CPS, Composite positive score; MSI, Microsatellite instability; MMR, Mismatch repair; MSS, MSI-low/microsatellite stability; pMMR, Proficient MMR; MSI-H, MSI-high; dMMR, Deficient MMR; LDH, Lactate dehydrogenase; ULN, Upper limit of normal; HER2,Human epidermal growth factor receptor 2; HR, Hazard ratios; CI, confidence intervals.

**Table 4:** Univariate and multivariate analysis of biomarkers for OS.

Variables	Univariate analysis		Model 1		Model 2	
	HR(95%CI)	*P*	HR(95%CI)	*P*	HR(95%CI)	*P*
Age						
>=65	Ref					
<65	1.033(0.649-1.643)	0.892				
Gender						
Male	Ref					
Female	2.146(1.363-3.379)	<0.001	2.691(1.514-4.782)	<0.001	3.384(1.747-6.555)	<0.001
ECOG						
0						
1	1.140(0.705-1.843)	0.593				
BMI (kg/m2)						
>=23	Ref					
<23	1.073(0.692-1.666)	0.752				
Gastrectomy						
No	Ref					
Yes	1.554(1.006-2.398)	0.047	2.011(1.129-3.581)	0.018	2.170(1.125-4.184)	0.021
Tumor location						
Non-GEJ	Ref					
GEJ	1.868(0.804-4.337)	0.146				
Tumor differentiation						
Poor	Ref					
Well-moderate	1.568(0.744-3.306)	0.237				
Lymph metastasis						
Yes	Ref					
No	1.050(0.641-1.719)	0.847			1.628(0.819-3.237)	0.165
Liver metastasis						
Yes	Ref					
No	0.921(0.582-1.457)	0.724			0.710(0.390-1.289)	0.26
Peritoneal metastasis						
No	Ref					
Yes	1.159(0.755-1.779)	0.5			0.582(0.295-1.149)	0.119
Chemotherapy regimens						
SOX/XELOX	Ref					
AS	1.001(0.543-1.847)	0.997			1.003(0.411-2.449)	0.995
Second-line						
No	Ref					
Yes	1.558(0.992-2.447)	0.054			1.202(0.629-2.297)	0.577
HER2 status						
Null	Ref					
Low	1.206(0.782-1.861)	0.397				
MSI status						
MSI-H/pMMR	Ref					
MSS/dMMR	1.954(0.597-6.393)	0.268				
CPS						
>=5	Ref					
<5	2.271(1.301-3.965)	0.004	1.943(1.106-3.413)	0.021	2.178(1.217-3.899)	0.009
LDH						
<=ULN	Ref					
>ULN	1.288(0.681-2.436)	0.436				
LDH increased						
<26%	Ref					
>=26%	2.689(1.657-4.362)	<0.001	4.254(2.186-8.279)	<0.001	4.380(2.228-8.614)	<0.001

ECOG, Eastern Cooperative Oncology Group; BMI, Body mass index; GEJ, Gastroesophageal junction; XELOX/SOX, Oxaliplatin +Capecitabine/Oxaliplatin+S-1; AS, Nab-paclitaxel+S-1; HER2,Human epidermal growth factor receptor 2; CPS, Composite positive score; MSI, Microsatellite instability; MMR, Mismatch repair; MSS, MSI-low/microsatellite stability; pMMR, Proficient MMR; MSI-H, MSI-high; dMMR, Deficient MMR; LDH, Lactate dehydrogenase; ULN, Upper limit of normal; HER2,Human epidermal growth factor receptor 2; HR, Hazard ratios; CI, confidence intervals.

Subsequently, the relationship between dynamic LDH changes and prognosis was further evaluated across subgroups stratified by different clinical characteristics. A post-treatment LDH increase ≥26% was consistently identified as a risk factor for survival(HR>1), a trend observed throughout the entire cohort. This association remained significant both in patients with liver metastasis and in those without liver metastasis (**Supplementary Figure 1**).

### 3.5 Composite biomarkers

**Figure 4:**
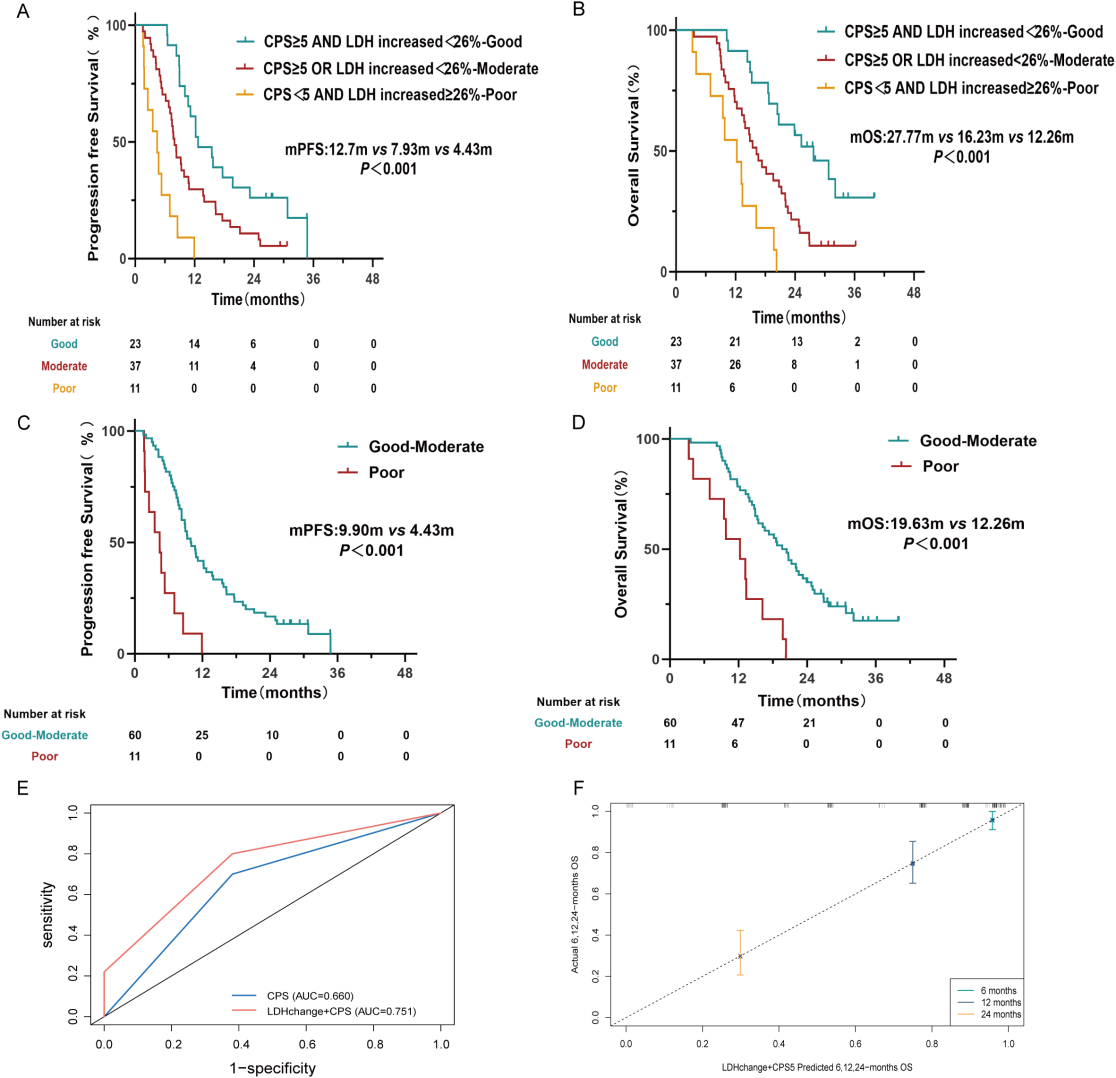
Kaplan-Meier survival curves for Progression-free survival (PFS) and Overall survival (OS) stratified by the composite biomarkers. **(A, B)** PFS and OS are stratified by combined CPS and LDH kinetics. Patients were categorized into three groups: CPS≥5 and LDH increase<26% (Good), CPS≥5 or LDH increase<26% (Moderate), and CPS<5 and LDH increase ≥26% (Poor). **(C, D)** PFS and OS comparing the combined Good-Moderate groups versus the Poor group. **(E)** ROC curve illustrating the comparative predictive performance of CPS alone versus CPS combined with LDH change for 2-year OS. **(F)** The calibration plot for the CPS combined with LDH change.

PD-L1 expression was evaluated in 71 patients, with 43 individuals (60.6%) demonstrating a CPS<5. Survival analysis revealed significantly prolonged PFS and OS in patients with CPS ≥5 compared to those with CPS<5 (P< 0.05) (**Supplementary Figure 2**). To assess the collective prognostic significance of PD-L1 CPS and post-treatment LDH dynamics, patients were stratified into three groups based on CPS and relative LDH change: Good (CPS≥5 and LDH increase<26%, n=23), Moderate (CPS≥5 or LDH increase<26%, n=37), and Poor (CPS<5 and LDH increase ≥26%, n=11). Substantial disparities in survival outcomes emerged across the groups. The median PFS was 12.7 months for the Good group, 7.93 months for the Moderate group, and 4.43 months for the Poor group (P< 0.001; **Figure 4A**). Consistently, the median OS across the three groups was 27.77, 16.23, and 12.26 months, respectively (P< 0.001) (**Figure 4B**). Combining the Good and Moderate groups (n=60, 84.5%) for comparison with the Poor group (n=11, 15.5%) demonstrated significantly longer median PFS (median 9.9 vs. 4.43 months, P< 0.001) and OS (median 19.63 vs. 12.26months, P< 0.001) (**Figures 4C, D**). ROC curve analysis indicated that the composite biomarker (LDH change + CPS) exhibited enhanced sensitivity and specificity for survival prediction compared to PD-L1 CPS alone, as indicated by a higher AUC for 2-year OS (0.751 vs 0.660) (**Figure 4E**). The calibration curve also demonstrates that the combined markers exhibit good calibration (**Figure 4F**).

### 3.6 Relationship between genetic mutation and efficacy

**Figure 5:**
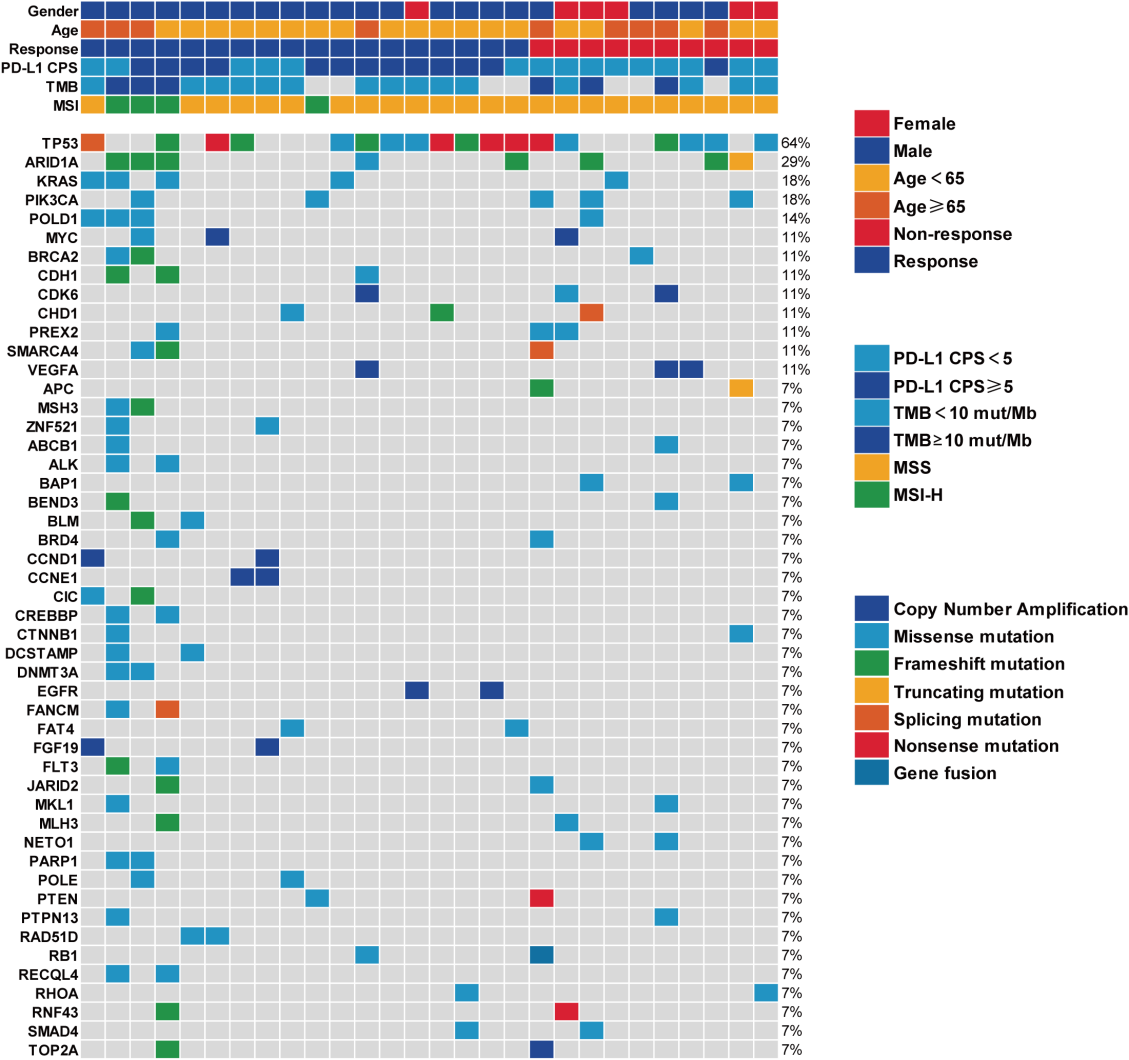
Distribution map of genetic mutation.

Genetic profiling was performed in 28 HER2-negative patients with advanced gastric cancer, revealing elevated mutation frequencies in TP53 and ARID1A (**Figure 5**). In the subgroup with a post-treatment LDH increase of<26%, a higher prevalence of KRAS mutations was identified. Exploratory analysis of KRAS mutation subtypes suggested that responses were more prevalent in individuals with KRAS G12C or G13D mutations, particularly when co-occurring with MSI-H or TP53 mutations, while patients with the KRAS G12D mutation showed no response (**Supplementary Table 6**). Notably, these findings are derived from a limited number of patients and warrant careful interpretation.

## 4 Discussion

The Tumor Microenvironment (TME) undergoes dynamic temporal changes, evolving throughout therapeutic interventions. Nevertheless, the practical limitations of serial tissue biopsies impede the real-time monitoring of these alterations. In contrast, longitudinal profiling of blood based biomarkers presents a clinically practical alternative, overcoming the challenges associated with repeated tissue sampling. Previous studies have demonstrated that dynamic modifications in peripheral blood biomarkers serve as indicators of treatment response in GC patients and are significantly correlated with prognosis. For example, reductions in the NLR, C-reactive protein-to-albumin ratio (CAR), PLR, and carbohydrate antigen 19-9 (CA19-9) levels post-treatment are strongly associated with prolonged PFS and OS in GC patients undergoing immunotherapy (14, 15). The integration and comprehensive analysis of patients’ clinical characteristics and hematologic parameters for treatment guidance and prognostic predictions represent crucial avenues for future research (16, 17). Our study has established dynamic LDH monitoring as a clinically actionable predictor of immunochemotherapy outcomes in HER2-negative AGC. Specifically, we have identified and validated a threshold of ≥26% increase in LDH as predictive of therapeutic response, marking the first such benchmark in gastric cancer. Sensitivity analysis was performed to evaluate the robustness of this threshold. Notably, a similar cutoff (≥25% LDH rise) has demonstrated prognostic significance in melanoma patients receiving immunotherapy (18), suggesting potential cross-cancer applicability of LDH dynamics.

Tumor cells commonly exhibit enhanced glycolytic metabolism, producing large amounts of lactic acid even under aerobic conditions. This metabolic shift is largely regulated by LDH, which catalyzes the conversion of pyruvate to lactate. Elevated systemic LDH levels often indicate heightened tumor glycolytic activity and can significantly impact the tumor immune microenvironment (19). On one hand, lactic acid, a byproduct of LDH activity, can acidify the TME, inhibiting the activity and function of cytotoxic T cells, natural killer cells, and other immune cells, thereby impairing the immune system’s ability to eliminate tumor cells. On the other hand, the acidic environment may promote the infiltration and functional enhancement of immunosuppressive cells, such as regulatory T cells and myeloid-derived suppressor cells, further suppressing the anti-tumor immune response. Additionally, lactate itself can directly modulate signaling pathways such as HIF-1α activation and G-protein-coupled receptor signaling, promoting tumor invasion and immune evasion (20, 21). Numerous studies have identified LDH as an independent prognostic biomarker across multiple cancer types (18, 22, 23). Dynamic changes in LDH levels may signify metabolic reprogramming in response to therapy. Our data align with previous findings in gastric cancer neoadjuvant settings, where stable LDH levels correlated with pathological response, highlighting its value as a metabolic sentinel capable of reflecting real-time alterations in tumor burden and microenvironmental stress (24).

Notably, the subgroup with poor prognosis (LDH increase ≥26% and CPS<5) exhibited a median OS of only 12.26 months (P<0.001), emphasizing the necessity for early alternative intervention strategies in this population. Furthermore, this strategy overcomes inherent limitations of standard imaging assessments. Radiologic responses may exhibit delayed manifestation, and pseudoprogression—an occurrence associated with immunotherapy-induced inflammatory reactions—can manifest in certain patients (25, 26). Regular dynamic evaluations every two treatment cycles enable precise identification of patients experiencing sustained benefit from immunochemotherapy. This approach allows timely detection of disease progression and subsequent therapeutic regimen optimization. Such a strategy not only mitigates the substantial time and economic costs associated with ineffective treatment but also significantly enhances therapeutic precision and efficacy.

In the subgroup of patients with a post-treatment increase in LDH of less than 26%, a higher prevalence of KRAS mutations was observed, which may be linked to favorable responses to immunotherapy. Specifically, patients carrying KRAS G12C or G13D mutations exhibited more favorable treatment responses, while those with KRAS G12D mutations did not show significant responses. Oncogenic KRAS signaling broadly influences tumor cell metabolism, affecting the utilization of glucose, lipids, and amino acids, and micropinocytosis (27). Furthermore, distinct KRAS mutation subtypes activate specific downstream signaling pathways, leading to divergent biological behaviors and immune microenvironment characteristics, potentially explaining the varied responses to immunotherapy. Consistent with our findings, in non-small cell lung cancer (NSCLC) patients treated with ICIs, individuals with KRAS mutations demonstrated a significantly higher ORR compared to KRAS wild-type patients, with KRAS G12C mutations associated with superior immunotherapy responses (28), while KRAS G12D was linked to poorer outcomes (29). These differences may be related to variations in PD-L1 expression and T-cell infiltration. Furthermore, the co-mutation status exerts influence on prognosis and treatment sensitivity, particularly in the context of response to checkpoint inhibition (30). Patients harboring KRAS/TP53 co-mutations demonstrated higher PD-L1 expression, increased CD8+ T-cell infiltration, along with more favorable responses to ICIs, compared to those with KRAS mutations alone (30, 31). This observation may provide insight into the outcomes observed in our cohort. However, given the limited sample size within the relevant subgroup in this study, the aforementioned conclusions should be considered hypothesis-generating rather than definitive. Further extensive clinical cohort studies are essential to systematically unravel the regulatory mechanisms of different KRAS mutation subtypes—along with their co-mutation patterns—on the immune microenvironment and their impact on treatment responses.

This study has several limitations (1): The retrospective single-center design and limited sample size may introduce selection bias (2). The absence of a chemotherapy-only control group in the study, despite all patients receiving uniform immunochemotherapy, makes it challenging to delineate the specificity of LDH dynamics within an immunotherapy-specific context (3). Lactate dehydrogenase (LDH), a nonspecific enzyme, can be influenced by non-neoplastic factors such as liver injury, infection/inflammation, hemolysis, and muscle damage. Despite adherence to standardized testing and pre-analytical quality control, these confounders cannot be completely mitigated. Therefore, in clinical practice, the interpretation of LDH level dynamics necessitates a comprehensive assessment in the context of the patient’s clinical profile to exclude alternative causes (4). Although the ≥26% increase in LDH was identified as a pivotal threshold and supported by sensitivity analyses, independent validation in a larger cohort is essential to affirm its clinical applicability (5). Peripheral blood biomarkers may not comprehensively capture the dynamic changes in the tumor microenvironment (6). Genomic analyses were substantially constrained by the small sample size, thus necessitating cautious interpretation and highlighting the need for further investigation.

Despite these considerations, our study proposes a novel strategy centered on dynamic biomarker monitoring. Given the evolving tumor microenvironment, serial biomarker assessment captures treatment-related biological changes more accurately and promptly than static measurements. Specifically, the composite biomarker developed here—combining baseline CPS with early LDH changes—can be calculated after two treatment cycles, positioning it as a practical tool for early risk stratification rather than for baseline treatment selection. Patients classified as having a poor prognosis (baseline CPS<5 and ≥26% increase in LDH after two cycles) demonstrate a higher likelihood of attenuated early response and greater disease aggressiveness. This stratification can guide clinical management by prompting intensified monitoring, earlier imaging and symptom assessment, timely treatment revaluation, and rapid multidisciplinary review to optimize subsequent therapy. For the group with a favorable prognosis, it may suggest a good response to the current immune checkpoint inhibition combination, and maintenance treatment may be considered, with a reduced frequency of efficacy assessment. However, these findings need to be further validated in a larger prospective multicenter cohort to confirm these findings and promote their application in clinical decision-making.

## 5 Conclusions

This study proposes and validates a novel risk stratification strategy utilizing dynamic biomarkers. In patients with HER2-negative advanced gastric cancer, an increase in LDH of ≥26% after two treatment cycles serves as an independent risk factor. By integrating baseline CPS with early on-treatment LDH dynamics, the resulting composite biomarker effectively identifies a poor-prognosis subgroup characterized by inferior early treatment response and more aggressive disease behavior. This tool delivers prognostic information at an early treatment stage, thereby guiding clinical monitoring and timely treatment revaluation to enable more personalized patient management.

## Data Availability

The raw data supporting the conclusions of this article will be made available by the authors, without undue reservation.
