# Supplementary material for: A composite biomarker based on early dynamic changes in lactate dehydrogenase and PD-L1 expression enhances outcome prediction for immunochemotherapy in HER2-negative advanced gastric cancer
Source: Front Immunol. 2026 Feb 12;17:1659007. doi: 10.3389/fimmu.2026.1659007 (PMC12935977; doi:10.3389/fimmu.2026.1659007)
Supplement: Supplementary file 1 [file Table1.docx]

Supplementary Material

# Supplementary Figures and Tables

## Supplementary Figures

**Supplementary Figure 1.** Subgroup analysis of the impact of lactate dehydrogenase (LDH) increase ≥26% versus <26% on PFS (A) and OS (B).


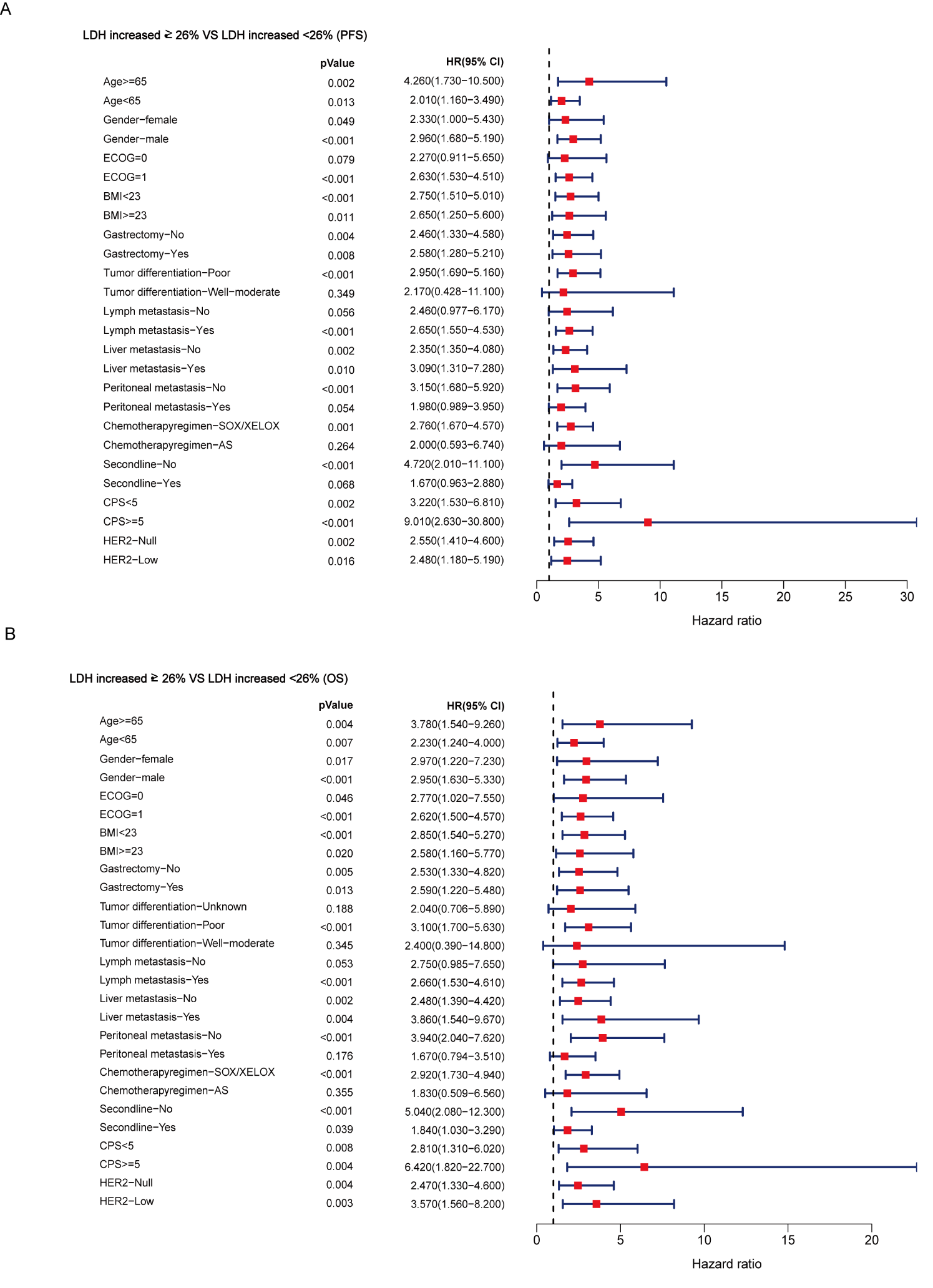


**Supplementary Figure 2.** Kaplan-Meier survival curves for PFS (A) and OS (B) stratified by the PD-L1 CPS in HER2-negative Gastric Cancer


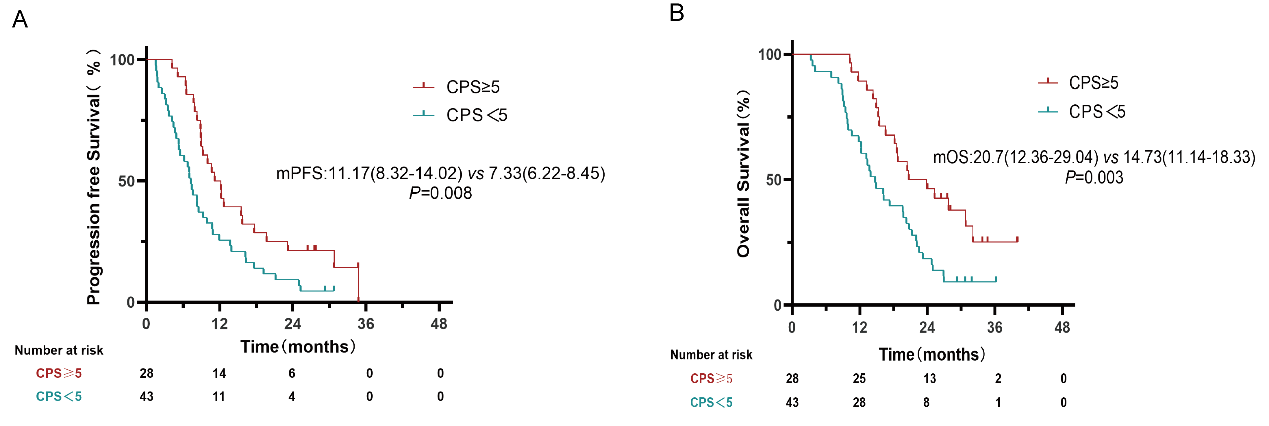


## Supplementary Tables

**Supplementary Table 1:** The detailed treatment plans

| **Component** | **Agents/Dosing** | **Cycle Duration** |
| --- | --- | --- |
| **Anti-PD-1** | Sintilimab (200 mg), Camrelizumab (200 mg), Toripalimab (240 mg), Pembrolizumab (200 mg), or Nivolumab (360 mg) | Q3W |
| **Chemotherapy** | **XELOX**: Oxaliplatin 130 mg/m² D1 + Capecitabine 850-1250 mg/m² BID D1-14 **SOX**: Oxaliplatin 130 mg/m² D1 + S-1 40-60 mg BID D1-14 **AS**: Nab-paclitaxel 130 mg/m² D1,8 + S-1 40-60 mg BID D1-14 | Q3W |

**Supplementary Table 2:** Formulas for the calculation of biomarkers

| **Biomarker** | **Full Name** | **Calculation Formula** |
| --- | --- | --- |
| CPS | Combined Positive Score | (Number of PD-L1-positive tumor cells + PD-L1-positive tumor-infiltrating immune cells) ÷ Total tumor cell count × 100 |
| NLR | Neutrophil-to-Lymphocyte Ratio | Neutrophil count ÷ Absolute lymphocyte count |
| LMR | Lymphocyte-to-Monocyte Ratio | Absolute lymphocyte count ÷ Monocyte count |
| PNI | Prognostic Nutritional Index | Serum albumin level (g/dL) + (5 × Absolute lymphocyte count) |

**Supplementary Table 3:** Hematological Biomarkers at Baseline and Post-Treatment

|  | **Baseline value Median (IQR)** | **Post-treatment value Median (IQR)** | **Percentage change Median (IQR)** |
| --- | --- | --- | --- |
| **NLR** | 2.5 (1.59, 3.71) | 2.07(1.49, 3.23) | -0.15 (-0.38, 0.24) |
| **LMR** | 3.31 (2.16, 4.56) | 2.97(2.14, 4.21) | -0.11 (-0.3, 0.34) |
| **PNI** | 48 (43.75, 51.22) | 47.30(43.85, 50.75) | -0.01 (-0.06, 0.06) |
| **WBC** | 6.13 (4.94, 7.46) | 5.53(4.30, 7.03) | -0.14 (-0.32, 0.16) |
| **LYM** | 1.63(1.20, 2.02) | 1.50(1.03, 2) | -0.06 (-0.21, 0.23) |
| **NEU** | 3.78 (2.64, 4.97) | 3.38(2.34, 4.43) | -0.16 (-0.38, 0.22) |
| **MONO** | 0.49 (0.36, 0.65) | 0.51(0.41, 0.68) | 0.08 (-0.22, 0.38) |
| **RBC** | 4.31(3.9, 4.67) | 3.93(3.59, 4.35) | -0.1 (-0.15, -0.03) |
| **HGB** | 127(111, 138) | 119(106, 131) | -0.05 (-0.11, 0.02) |
| **PLT** | 232 (180, 285.5) | 191(136, 238) | -0.13 (-0.36, 0.06) |
| **PCT** | 0.23(0.18, 0.28) | 0.18(0.13, 0.22) | -0.16 (-0.39, 0.03) |
| **ALT** | 15 (11, 23) | 16(12, 25) | 0.07 (-0.3, 0.5) |
| **AST** | 21 (17, 29.5) | 27(21, 36) | 0.17 (-0.12, 0.52) |
| **ALP** | 102 (78, 131.5) | 95(77, 124) | -0.04 (-0.21, 0.15) |
| **LDH** | 180(158, 216) | 198(169, 247) | 0.06(-0.06, 0.27) |
| **TB** | 13.5 (9.65, 17.8) | 14.8(11.8,17.8) | 0.13 (-0.19, 0.38) |
| **TBA** | 3 (1.81, 6.72) | 4.10(1,.91, 9.51) | 0.27 (-0.37, 1.49) |
| **ALB** | 39.5 (37.25, 42) | 39.2(35.9, 43) | 0 (-0.06, 0.05) |
| **GLOB** | 31.8(28.6, 35.8) | 31.7(26.8, 35.8) | -0.01(-0.10, 0.08) |
| **CEA** | 4.03 (1.92, 11.36) | 4.25(1.91, 8.92) | 0.04 (-0.44, 0.53) |
| **CA199** | 21.58 (8.74, 102.31) | 19.81(8.55, 45.41) | -0.08 (-0.6, 0.26) |

NLR: neutrophil-to-lymphocyte ratio; LMR: lymphocyte-to-monocyte Ratio; PNI: prognostic nutritional index; WBC: white blood cell; LYM: lymphocyte; NEU: neutrophil; MONO: monocyte; RBC: red blood cell; HGB: hemoglobin; PLT: platelets; PCT: platelet crit; ALT: alanine transaminase; AST: aspartate transaminase; ALP: alkaline phosphatase LDH: lactate dehydrogenase; TB: total bilirubin; TBA: total bile acid; ALB: albumin; GLOB: globulin; CEA: carcinoembryonic antigen; CA199:carbohydrate antigen 199;

**Supplementary Table 4**: Sensitivity Analysis of the Association Between Various LDH Increase Thresholds and Overall Survival

| **Cut-off Value (%)** | **Number of Patients (High Increase)**) | **Number of Patients (Low Increase)** | **Log-rank P Value** | **Hazard Ratio (HR)** |
| --- | --- | --- | --- | --- |
| 12% | 50 | 57 | 0.059 | 1.512 |
| 15% | 42 | 65 | 0.08 | 1.481 |
| 18% | 38 | 59 | 0.031 | 1.638 |
| 21% | 35 | 72 | 0.001 | 2.067 |
| 24% | 31 | 76 | **<0.001** | 2.481 |
| **26%** | **29** | **78** | **<0.001** | **2.689** |
| 28% | 26 | 81 | **<0.001** | 2.506 |
| 31% | 21 | 86 | **0.001** | 2.456 |
| 34% | 21 | 86 | **0.001** | 2.456 |
| 37% | 19 | 88 | **<0.001** | 2.933 |
| 40% | 18 | 89 | **<0.001** | 3.006 |

**Supplementary Table 5**: Baseline characteristics of 107 patients stratified by dynamic LDH change after treatment

| **Variables** | **Total**  **(n = 107)** | **LDH＜26% increased**  **(n = 78)** | **LDH≥26% increased**  **(n = 29)** | **P value** |
| --- | --- | --- | --- | --- |
| **Age, n (%)** |  |  |  | 0.793 |
| <65 | 74 (69) | 55 (71) | 19 (66) |  |
| ≥65 | 33 (31) | 23 (29) | 10 (34) |  |
| **Gender, n (%)** |  |  |  | 0.935 |
| Female | 32 (30) | 24 (31) | 8 (28) |  |
| Male | 75 (70) | 54 (69) | 21 (72) |  |
| **ECOG, n (%)** |  |  |  | 0.965 |
| 0 | 28 (26) | 21 (27) | 7 (24) |  |
| 1 | 79 (74) | 57 (73) | 22 (76) |  |
| **BMI (kg/m2), n (%)** |  |  |  | 0.862 |
| <23 | 66 (62) | 49 (63) | 17 (59) |  |
| ≥23 | 41 (38) | 29 (37) | 12 (41) |  |
| **Gastrectomy, n (%)** |  |  |  | 0.346 |
| No | 65 (61) | 50 (64) | 15 (52) |  |
| Yes | 42 (39) | 28 (36) | 14 (48) |  |
| **Tumor location, n (%)** |  |  |  | 1 |
| GEJ | 6 (6) | 5 (6) | 1 (3) |  |
| Non-GEJ | 101 (94) | 73 (94) | 28 (97) |  |
| **Tumor differentiation n (%)** |  |  |  | 0.821 |
| Unknown | 25 (23) | 19 (24) | 6 (21) |  |
| Poor | 74 (69) | 54 (69) | 20 (69) |  |
| Well-moderate | 8 (7) | 5 (6) | 3 (10) |  |
| **Lymph metastasis, n (%)** | 78 (73) | 56 (72) | 22 (76) | 0.86 |
| **Liver metastasis, n (%)** | 33 (31) | 25 (32) | 8 (28) | 0.834 |
| **Peritoneal metastasis, n (%)** | 46 (43) | 34 (44) | 12 (41) | 1 |
| **Chemotherapy regimens, n (%)** |  |  |  | 1 |
| SOX/XELOX | 92 (86) | 67 (86) | 25 (86) |  |
| AS | 15 (14) | 11 (14) | 4 (14) |  |
| **Second-line, n (%)** |  |  |  | 0.402 |
| No | 42 (39) | 33 (42) | 9 (31) |  |
| Yes | 65 (61) | 45 (58) | 20 (69) |  |
| **HER2 status, n (%)** |  |  |  | 1 |
| Null | 67 (63) | 49 (63) | 18 (62) |  |
| Low | 40 (37) | 29 (37) | 11 (38) |  |
| **CPS, n (%)** |  |  |  | 0.254 |
| <5 | 43 (40) | 32 (41) | 11 (38) |  |
| >=5 | 28 (26) | 23 (29) | 5 (17) |  |
| Unknown | 36 (34) | 23 (29) | 13 (45) |  |
| **MSI status, n (%)** |  |  |  | 0.756 |
| MSS/ dMMR | 46 (43) | 34 (44) | 12 (41) |  |
| MSI-H/ pMMR | 5 (5) | 3 (4) | 2 (7) |  |
| Unknown | 56 (52) | 41 (53) | 15 (52) |  |

ECOG: Eastern Cooperative Oncology Group; BMI: Body mass index; GEJ: Gastroesophageal junction; PD-L1: Programmed cell death protein ligand 1; CPS: Composite positive score; MSI: Microsatellite instability; MMR: Mismatch repair; MSS: MSI-low/microsatellite stability; pMMR: Proficient MMR; MSI-H: MSI-high; dMMR: Deficient MMR; LDH: Lactate dehydrogenase; ULN: Upper limit of normal; HER2:Human epidermal growth factor receptor 2;

**Supplementary Table 6:** Characteristics and survival of patients with KRAS-mutated gastric cancer

|  | **KRAS mutation site** | **MSI** | **TP53 mutations** | **PFS(months)** | **OS(months)** |
| --- | --- | --- | --- | --- | --- |
| Patient 1 | G12D | MSS | Wild-type | 4.9 | 10.5 |
| Patient 2 | G12C | MSI-H | Frameshift mutation | 31.63 | 31.63 |
| Patient 3 | G13D | MSI-H | Wild-type | 22.63 | 22.63 |
| Patient 4 | G12S | MSS | Splicing mutation | 26.5 | 26.5 |
| Patient 5 | G12D | MSS | Missense mutation | 6.53 | 20.7 |

KRAS: Kirsten rat sarcoma viral oncogene homolog; MSI: Microsatellite instability; MSI-H: MSI-high; MSS: MSI-low/microsatellite stability; TP53: Tumor protein 53; PFS: progression-free survival; OS: overall survival
